# Having allies—Experiences of support in people with stress-related exhaustion: A qualitative study

**DOI:** 10.1371/journal.pone.0277264

**Published:** 2022-11-09

**Authors:** Sara Alsén, Lilas Ali, Inger Ekman, Andreas Fors

**Affiliations:** 1 Institute of Health and Care Sciences, Sahlgrenska Academy, University of Gothenburg, Gothenburg, Sweden; 2 Centre for Person-Centred Care (GPCC), University of Gothenburg, Gothenburg, Sweden; 3 Department of Psychiatry, Sahlgrenska University Hospital, Gothenburg, Sweden; 4 Department of Medicine, Geriatrics and Emergency Medicine, Sahlgrenska University, Hospital/Ostra, Gothenburg, Sweden; 5 Region Västra Götaland, Research and Development Primary Health Care, Gothenburg, Sweden; Public Library of Science, UNITED STATES

## Abstract

**Background:**

The number of people seeking care for symptoms of exhaustion and stress is a major concern in several countries. The condition is a challenging and life-changing experience, and a deeper understanding of support to help people on sick leave due to stress-related exhaustion in their early stages is needed to facilitate recovery.

**Objective:**

The aim was to explore experiences of support in people with stress-related exhaustion being on sick-leave less than six months.

**Method:**

A qualitative interview study was conducted with 12 participants (7 women and 5 men; aged 25–46 years) who were on sick leave that had not exceeded six months due to stress-related exhaustion. The participants were recruited from public healthcare centres in the western part of Sweden, and the intention was to reach them early in their ongoing sick leave period. The interviews were performed face-to-face and analysed using a phenomenological hermeneutical approach.

**Results:**

The findings show that people affected by stress-related exhaustion struggle to maintain their dignity and define support in terms of allies who acknowledge their personhood and provide them helpful guidance. Participants expressed their needs to be taken seriously by professionals, family, and friends willing to engage in their situation. Lack of empathy and professional knowledge in the clinical encounter induced additional stress.

**Conclusions:**

This study indicates that people affected by stress-related exhaustion need allies from their surrounding networks in their struggle to maintain their dignity. Our findings highlight that it is essential to acknowledge them as persons and establish an alliance to provide appropriate support based on each person’s specific situation, needs and resources. This approach can be facilitated in a partnership, as emphasized in person-centred care (PCC). PCC emphasizes the co-creation of care in partnership between the patient (often with relatives) and health care professionals which may imply a more collaborative view of health care in which patients are engaged as active partners in planning their care.

## Introduction

The number of people seeking care for symptoms of exhaustion and stress is a major concern in several countries [1–3]. European sick leave figures show increasing numbers [4], and stress-related illness is the most frequent reason for sick leave in Sweden [5]. Exposure to stress has been shown to increase the risk for both physical [6–8] and mental [9–11] symptoms, and cognitive impairment is commonly reported [12]. Stress-related illness has also been shown to have a negative impact on the affected person’s family, and its costs to society in terms of healthcare and productivity loss for employers is high [13, 14]. Internationally, the term ‘burnout’ is often used to describe severe stress-related mental problems. Burnout is solely related to workplace stress and characterized by three dimensions: feelings of energy depletion or exhaustion, feelings of cynicism and detatchment from one’s job, and reduced job efficiency [15, 16]. The most commonly used burnout tool, the Maslach Burnout Inventory, does not seem suitable as a single diagnostic tool in clinical practice due to its high probability of overdiagnosing burnout [17].

To improve the diagnostics of stress-related exhaustion [18–21], an expert group at the Swedish National Board of Health and Welfare suggested specific diagnostic criteria for stress-related exhaustion aimed at patients with apparent mental and physical exhaustion due to prolonged stress [2]. These criteria resulted in the introduction of a new diagnosis, exhaustion disorder, F43.8A in the Swedish version of the International Classification of Diseases Version 10 (ICD-10) [22]. According to these criteria, exhaustion disorder is marked by a lack of psychological energy and the following symptoms: cognitive deficits, emotional instability, reduced ability to cope with demands and/or time pressure, disturbed sleep, physical weakness, and physical symptoms such as muscular pain. The symptoms need to be present for over six months and attributed to identifiable stressful events, work- or non work-related (such as private relational conflicts) [19–21, 23] that last for at least two weeks.

In the Governmental Social Insurance Agency report, exhaustion disorder is described as a separate basis for sick leave, with a recovery period which, even with specialized rehabilitation and a gradual return to work, is expected to extend ‘not infrequently more than six months and in some cases up to a year or more’ [24]. A long-term follow up study in Sweden showed that almost half of the patients with stress-related exhaustion report symtoms such as fatigue and that one third are clinically assessed with exhaustion seven years after initially seeking care [25]. Interview studies have found that stress-related exhaustion can be experienced as a loss of access to oneself and one’s context and feelings of being trapped and lost in life [26, 27]. The condition is a challenging, life-changing existential experience that can combine a crisis with an opportunity for new insights [26]. It is thus challenging to treat successfully, and existing evidence in rehabilitation models are limited. However, interventions that include a workplace component during rehabilitation are more successful in improving return to work [28–30].

There are many supporting factors associated with the recovery process, and it requires a well-organized support system from family, friends, healthcare professionals, primary care, public health, and social services [31].

According to the Swedish National Board of Health and Welfare [24], treatment and rehabilitation should be based on supporting sleep, reducing anxiety and facilitating a balance between activity and rest. Rehabilitation is recommended to include changes in lifestyle, stress management, and a gradual return to a manageable way of life [24]. Evaluations of rehabilitation programmes in Sweden identify being part of a group and feeling respected by others as essential to promoting health [32], recovery [33–35], and return to work [36, 37]. Being affected by stress-related exhaustion has a negative impact on patients’ health [25], increases the risk for stigmatisation [38], and often involves a longer period of sick leave in relation to other stress-related disorders [24]. Previous research has mainly focused on experiences of different treatment interventions and rehabilitation programmes [32–35]. Hence, a deepened understanding of support to people on sick leave due to stress-related exhaustion in the early stages of their sick-leave to manage their lives and regain their health is essential to reduce suffering and enhance recovery. Therefore, this study aimed to explore experiences of support in people with stress-related exhaustion on sick-leave less than six months.

## Method

### Design

A qualitative interview study was undertaken to meet the aim of the study. Data were analysed using a phenomenological hermeneutic approach inspired by Paul Ricoeur´s interpretation theory [39] and further developed for research by Lindseth & Norberg [40]. This approach is designed to interpret narratives as texts to obtain understanding and knowledge of people’s perspectives [39, 40]. Using Ricoeur’s phenomenological hermeneutics helped us understand the meaning of a particular phenomenon, in this study, the meaning of support when living with stress-related exhaustion. The study adheres to the Standards for Reporting Qualitative Research guidelines [41].

### Participants and setting

Participants were recruited from nine public primary healthcare centres in the western part of Sweden. A purposeful sampling strategy aimed to reach participants in the early stages of sick leave, resulting in a cohort of men and women of various ages, including white- and blue-collar employees. Patients were screened through medical records by designated healthcare professionals according to the following inclusion criteria: (a) diagnosed with exhaustion disorder, (b) on sick leave due to exhaustion disorder no longer than six months and (c) having the physical and mental capacity to participate in an interview. Potential participants were contacted by phone and asked if they wanted to participate. Patients willing to participate received written information about the study, and a date and time for the interviews were set. A total of 19 people were invited to participate, of which 3 declined, and 4 withdrew before the interview because they lacked sufficient energy to participate. Consequently, 12 people (7 women and 5 men aged 25–46 years) were included (Table 1). Their current percentage of sick leave ranged from 50% to 100% of work hours, except for one who resumed work two days before the interview.

**Table 1:** Demographic information of participants.

Participants	Female *n* = 7	Male *n* = 5
Age		
25–34	3	2
35–44	4	2
45–54		1
Civil status		
Living with partner	5	1
Living alone	2	4
Employee		
Blue collar	2	2
White collar	5	3

### Interviews

The first author (SA) conducted the interviews face-to-face from June 2018 to February 2019 in a location (participant’s home or workplace, first author’s workplace, or café) of the interviewees’ choosing and transcribed them verbatim. To encourage narration, the interviewer asked open-ended questions beginning with ‘How do you experience support in everyday life (when being affected by stress-related exhaustion)? Probing questions such as ‘How do you feel about that?’ and ‘What does that mean to you?’ were asked to evoke comprehensive responses. Additional questions were “What does support mean to you in your situation?”, and “Is there anything you would like to add”? The interview guide can be found in the S1 File. The interviews were performed 2 to 4 months after the sick leave began, lasted from 28 to 79 (median 45) minutes, and continued until the authors considered the research question answered in full.

### Ethical considerations

Approval for the study was obtained from the Ethical Review Board at the University of Gothenburg (Dnr: 497–17, T 526–18). Participants were given written and oral information about the study and gave their written consent to participate. The authors were aware that participants might be affected by sharing their thoughts and experiences, and if so, they were offered consultation with a counsellor. All ethical decisions were guided by the World Medical Association Declaration of Helsinki [42].

### Interpretation of data

The interviews were analysed using a phenomenological hermeneutical method, inspired by Ricoeur´s theory of interpretation [39, 40]. This method intends to generate an understanding of the meaning of a particular phenomenon, in this case, experiences of support when living with stress-related exhaustion. The interpretation consists of three interrelated phases: **naïve reading, structural analysis, and interpretation of the whole** [39]. In the *naïve reading*, the narratives were read several times to form a first impression of the overall meaning of their contents. The *structural analysis*, conducted in several steps, confirmed the first impression of the naïve reading and identified sequences of the text relevant to the aim of the study. The authors discussed their overall impression of the text, the meaning units, and the interpretations of the text in several analyses. In the final part of the analysis, *interpretation of the whole*, the entire text was re-read and interpreted in relation to the *naïve reading*, *structural analysis* and the authors’ pre-understandings to formulate a comprehensive understanding of the narratives. The authors pre-understanding include clinical experience from psychiatric care (SA and LA), hospital care (IE) and primary care (AF). The research group has extensive experience in qualitative methods including phenomenological hermeneutics. The first two steps (*naïve reading* and *structural analysis*) will be presented in the findings, and the last step, *the interpretation of the whole*, is presented with references related to the area and the psychologist Carl Roger’s (1902–1987) theory on the therapeutic alliance [43]. According to Lindseth and Norberg, the interviewees can only understand and narrate their lived experiences in relation to their preunderstanding. The authors cannot free themselves from their preunderstanding when interpreting texts, and are only aware of aspects of it [40].

## Findings

### Naive understanding

The first impression of the interviews was that participants experienced support when they met people in their surrounding networks who understood and guided them. The surrounding network included professionals in healthcare and workplace representatives, as well as family and friends. A shared understanding of their situation was crucial, and the participants struggled to be acknowledged and maintain their dignity as persons. Feeling acknowledged depended upon the benevolence and ability of people in their surrounding networks to understand stress-related exhaustion in light of the participant’s life situation. Finding persons willing to engage in their rehabilitation reduced stress and made the participants feel dignified, which facilitated the start of their recovery. The absence of acknowledgment increased stress and obstructed their opportunities to find new routines and paths in life.

### Structural analysis

In the structural analysis the text was first divided into meaning units conveying the essential meaning of the lived experiences. These units were formulated as condensed descriptions in a way that disclosed the meaning and critically reflected upon in comparison with the naïve understanding. Meaning units with similar content were divided into groups, which were abstracted to form subthemes, which were further abstracted to formulate themes. The meaning of support is formulated in the main theme, **Having allies to maintain one’s dignity**. The main theme was developed from the themes **Being acknowledged** and **Proper guidance**. These themes emerge from six subthemes presented in relation to each theme and illustrated by quotations (Table 2).

**Table 2:** Main theme, theme, and subthemes.

Main Theme: Having allies to maintain one´s dignity	
Theme:	Subtheme:
Being acknowledged	Being taken seriously
	Personal commitment
	Time for recovery
Proper guidance	Qualified and trustworthy professionals
	Tailored interventions
	Being trusted to establish new routines

#### Being acknowledged

Feeling supported in everyday life meant being seen by people in their surrounding networks as persons first and not only as a diagnosis that is associated with impairments. This was expressed in three subthemes: being taken seriously, personal commitment, and time for recovery.

**Being taken seriously** meant that people in their surrounding networks took the time to listen to the participants. The participants emphasized that being listened to made it easier for them to relax and express their feelings. Being able to narrate their experiences facilitated a shared understanding of the situation. The participants also became more open to information and other perspectives from these persons. Professionals who listened carefully were perceived to care for them and be compassionate, making participants feel unique and respected, rather than just a patient or employer in line for a cure.

That the person [a physician] proceeds based on my situation, how I am experiencing it. Not to proceed based on the diagnosis, but rather that of course, she [the physician] understands that I have this diagnosis–but she proceeds based on how I am experiencing it, rather than the reverse. Really listens to me. To be seen as an individual and not as a patient. (5)

The importance of a shared understanding with friends is exemplified in the quote below.

She [a friend] sees my struggling up close. She knows and sees it, and then she sees how I feel too. She knows how I feel. She is, just like me a very sensitive and strong person. We capture emotions very easily. So she sees me. (6)

Feeling able to express their thoughts and concerns about their experiences without being questioned made the participants feel less lonely and helpless. Professionals with an open and accepting approach were perceived as trustworthy, and the opposite approach obstructed their recovery.

It feels wrong to not be taken seriously [physician and manager]. It feels absolutely devastating, or for me it has made me feel a whole lot worse, and made me lose confidence in my physician and the healthcare system… the feeling that something is wrong, no one believes me. That made it so hard to then try to get healthy by resting, just thinking that no one believes me. (9)

**Personal commitment** meant that the people in their surrounding networks took a personal interest in the participants’ situation and made genuine efforts to ease the participants’ burden, shown through extended care in addition to everyday friendship or outside professional responsibilities. Professionals who made extra telephone calls and were willing to extend or rebook appointments contributed to building a personal relationships, and their information and advice were perceived as emphatic and credible.

She [the physician] called two, three times to ask how I was doing, how everything was working. She reminded me to go in to the accident and emergency department if it got worse… People [family and friends] around me care, but they can’t help me in the same way as healthcare people can. So it was brilliant to know that a person [the physician] who can actually help me cares enough to get in touch and just chat for 30 seconds. I wasn’t just a patient to her. (3)

The participants’ need for help and support often developed a bad conscience about their family and friends, which could evoke more negative feelings such as being lost. A supportive social network that could help ease these burdens was essential to support whose accepting participants’ acknowledgment reduced their feelings of shame and guilt.

It’s great to have support, that people [family and friends] support you and help you to remember things. But it gets more irksome with friends and acquaintances who think I’m just lazy. It gets really annoying, and then I just want to show them that I’m not lazy, so I try again. Then I get even worse. (4)

**Time for recovery** was necessary after a long time of high-paced life; participants required the opposite: a reduced tempo and time for recovery. Sick leave provided time to catch up with themselves, to focus on resting, and to minimise stressful stimuli. The participants needed time to figure out how to live their life.

I feel a strong need to catch up with myself, as I’ve not had time for myself at all. That has been my lowest priority, so I need to reassess my entire situation. Now I need to put myself first, so that I can keep it together… I need the time to figure out what the hell I’m doing. So the time aspect feels important to me. (5)

Sick leave was a crucial part of their recovery in which they depended on a positive approach and understanding about their situation from health care professionals. Not being understood and cared about obstructed their support and recovery.

Damn, you’ve tried to process this for a long time with the same physician, who has been nice, let you be on sick leave, taken it easy. And then you get a new physician who just says; ‘Well, you know that this is now the fifth month you’ve been on sick leave, unfortunately I can’t sick-list you any longer’.(11)

**Proper guidance** was interpreted as the participants need for practical guidance, which was expressed in the following subthemes: qualified and trustworthy professionals, tailored interventions, and being trusted to establishing new routines.

**Qualified and trustworthy professionals** who understood the complexity of stress-related exhaustion were essential to participants seeking support, treatment, and advice. Professionals who understood the importance and power of a personal, but still professional, relationship based on knowledge and empathy, who listened to participants’ narratives and experiences, contributed to a supportive relationship and made participants feel they were in safe hands and could trust professionals to help them.

She [workplace representative] is outstanding. She has been exhausted, so she has just pressed the brake for me. She says, "now we do this", and she is really supportive. I am lucky not to have an employer who calls and stresses you to come back and has always been like caring instead of pushing or being suspicious. It has been a factor that enabled me to cope with this. (9)

Knowledge of stress-related exhaustion and recommended treatment guidelines was a prerequisite to adequate professional support. Professionals with limited knowledge of stress-related exhaustion and who did not meet the participants as unique persons were experienced as offensive and contributed to further stress.

I went to a meeting with a rehabilitation coordinator in primary care, and at that meeting, she thought I was ok. And it was hard because I felt the opposite. I told her, "I should not have to smear my hair in coconut oil for you to understand that I don’t feel well". Just trust what I say and listen to me". (3)

**Tailored interventions** with guidance helped participants who were drained of energy to prioritise, take decisions and move in the right direction. This could mean family and friends making decisions according to everyday activities and that professionals took command of deciding when to schedule a new appointment. This support spared some energy and allowed the participants to focus on themselves and create private and personal changes.

When you are at that stage [exhausted] and in the health care process, I should not have to say what I need. The first step (to get in contact with health care), is uncertain, and I have to take that. But once there, they should lead the way. (1)

Healthcare professionals who offered flexible interventions and tailored them to the participants’ needs were also crucial in their recovery. For example, the participants could participate in treatment groups based on their ability, which could include participation every other week instead of every week (recommended by the guidelines). Being forced into a ’one size fits all’ system obstructed the process. The participants appreciated when professionals customised beyond organisational rules and regulations. One participant described unflexible support when referring to the Social Insurance agency.

The Social Insurance Agency determines when and how one is to work, which feels completely idiotic. For me it is absolutely best to be off on a Monday, as then the week begins and, because I’m a person who, once things get going, it’s hard to put on the brakes. It is incredibly peculiar that they should sit there and judge such an aspect… It has to do enormously with the individual situation. (5)

*Being trusted to establish new routines*. Receiving a sick leave certificate and understanding from people in their surrounding networks instead of struggling for acknowledgment reduced stress and facilitated self-reflection and a chance to establish new routines and paths in their lives. Time off from work and obligations supported their reorientation in everyday life. The priorities were now to find more solid ground to stand on.

I try to distance myself from things that stress me too much. I try to see my limits and actually set them. My most extensive support is myself. I choose my battles with myself and also at my workplace. To have hope also helps. I will see my physiotherapist and have been contacted by another clinic for my mental state. There is hope as well. (3)

Prioritizing themselves was unusual, and time off with additional support from people in their surrounding networks facilitated energy and motivation to healthy activities, such as exercise and being with friends. Paying attention to their body’s signals helped participants acknowledge their boundaries and focus on themselves and their needs.

I work out about five days a week, but I also try to listen to my body. Yesterday I didn’t feel like it after work, and instead I went home and slept for two hours. It’s no longer about me having to perform all the time, but it’s okay if I go home, sleep, and even talk about it with someone else. Before I felt like I couldn’t say that I was just staying home and not doing anything. (7)

### Interpretation of the whole

As described by Lindseth and Norberg [40] theoretical concepts are recommended to be included in the third step in order to enrich and deepen the understanding of the data. Hence, in this step of the analysis (interpretation of the whole) we have consulted our preunderstanding, literature and considered the concept of the terapeutic alliance according to Carl Rogers [43]. Our overall interpretation is that people affected by stress-related exhaustion seem to be in a struggle to maintain their dignity. Having support means having allies in this struggle.

The findings emphasize the importance of having allies in the struggle with the consequences of stress-related exhaustion, but also reveal the participants’ vulnerability when they do not feel acknowledged and respected from people in their surrounding networks (e.g. professionals [healthcare professionals, work-place representatives] and family and friends). Falling ill and requiring support were experienced as a threat to the participants’ dignity and their value as a person. Having allies that acknowledged the participants as persons and showed them respect made them respect themselves and feel dignified. In the participants’ struggle for dignity and acknowledgment, they experienced support as having allies, defined as people in their surrounding networks engaged with them in a mutually respectful relationship to achieve a shared understanding and agreement. The findings highlight the importance of an interpersonal relationship in which people affected by stress-related exhaustion and people in their surrounding networks join as allies. To further deepen the understanding of the meaning of support when being affected by stress-related exhaustion, this foundation and its prerequisites can be understood as a therapeutic alliance [44, 45], as described by Carl Rogers. According to Rogers, a therapeutic alliance consists of three core elements: empathy, congruence (genuineness), and unconditional positive regard (acceptance) [45]. Empathy is closely related to sympathy, which is congruent with the theme *Being acknowledged* and highlights the importance of being treated as a person–almost a friend–not just a patient. Genuineness is required to create a safe, warm, and tolerant environment, congruent with the subthemes *Qualified and trustworthy professionals* and *Personal commitment* and highlighting the importance of a personal and emotional engagement in clinical encounters. According to Rogers, acceptance means that the people set aside their personal opinions, which is similar to the subtheme *Being taken seriously* and emphasizes the importance of giving the participants time and space to tell their illness stories without interruption or questions. Not being forced into ‘one size fits all’ as described in the subtheme *Tailored interventions* further indicates the need for professionals to provide support beyond their personal opinions and recommended guidelines.

According to Rogers, professionals need to accept the client as a person and see the world through their eyes [44]. Even though healthcare professionals strive to do their uttermost to support patients, they need to follow organisational rules and regulations where it is difficult to be flexible [46, 47]. When in contact with healthcare, we found that the participants struggled against the feeling of being reduced to one’s disease and the effort to maintain the notion of oneself as a person. In encounters with professionals, the participants depended on their ability to understand and care for them. Being treated as a person is also highlighted by the Swedish philosopher Fredrik Sveneaus [48], who considers it unethical to not view patients as persons. An objectifying approach towards the patients’ condition increases the risk of missing valuable information about the condition’s subjective experience [48]. The participants in our study described feeling seen and treated as persons when their family and friends extended their care in addition to the everyday relationship, and professionals extended their actions beyond the call of duty. Actions or small gestures such as additional telephone calls, visits, or kind words reduced the participants’ risk of feeling objectified. This assumption is congruent with Topor et al. [49], who highlight the importance of ‘small things’ in improving the quality of care. Such small spontaneous signs in professional practice convey a message of shared humanity and hope, affect a person’s sense of self, and remind people of themselves as persons rather then patients. When professionals go beyond the boundaries of the professional role, a friendly relationship occurs. They act and relate as friends [49], congruent with our theme *being acknowledged*, emphasizing the importance of being treated not just as a patient but as a person–almost a friend.

We interpreted encounters in which professionals did not meet the person but rather the disease or had insufficient knowledge of stress-related exhaustion as inadequate support. The participants needed *Proper guidance* to receive that kind of support, and professionals needed to get to know the participants to understand the challenges, needs, and complexity associated with stress-related exhaustion. Bolton [50], described this challenge for mental healthcare professionals, who face interpretation challenges within the diagnostic biomedical framework of health and disease. Clinical diagnoses, such as stress-related exhaustion, are based on signs, symptoms, and disability that are not considered ‘normal’, and the distinction between normal and pathological reactions is difficult. As our findings and previous research show that people affected by stress-related exhaustion suffer from shame and stigmatisation due to their condition, which also raises existential issues [26, 38], it is essential to acknowledge descriptions of symptoms and consider lived experiences in the interpretation process [50]. The interpretation of our findings made it clear that having allies facilitated the participants’ ability to self-support. Rogers, asserted the therapeutic alliance as an equal partnership, in which the therapist helps people to find their own solutions, increasing empowerment and enabling change [43, 44]. The power of having allies in the struggle with stress-related exhaustion is thereby obvious, and as is the power of an absence of allies to obstruct recovery and possibly even worsen symptoms of the disease. The findings also showed that professionals need to engage on a personal level beyond their professional role to support people with stress-related exhaustion.

Lack of empathy, genuineness, and acceptance in clinical encounters contributed to additional challenges for the participants. Instead of dealing with their illness, they struggled for acknowledgment and dignity. A previous study conducted later in the rehabilitation process underlined the importance of being respected and listened to [32], and unsupportive encounters have been shown to increase distress in patients with stress-related exhaustion [19, 51]. The absence of acknowledgment has also been shown to contribute to detachment from the body [27] and existential anxiety [26]. Informants diagnosed as burned out reported that when they felt distrusted, they no longer knew themselves [51]. Hence, it is essential to acknowledge and respect the person seeking help for stress-related exhaustion to minimize negative feelings, reduce symptoms, and improve recovery. It is essential that professionals be aware of the complexity of stress-related exhaustion, the importance of the interpersonal relationship, and people’s subjective experiences as decisive components in supporting these people.

In summary, people in the surrounding networks to persons affected by stress-related exhaustion need to acknowledge the person beyond the disease and its impairments as the condition has a personal and existential impact. It is essential to listen thoroughly to the patients’ narratives and develop a shared understanding of their experiences, so the person feels heard and respected. This approach can be facilitated in a partnership and by using a jointly agreed health plan, as emphasized in person-centred care (PCC). A PCC approach in health care highlights the importance of knowing the patient as a person with resources and needs, understanding the illness from the patient’s perspective, and treating the patient as a person with autonomy and capabilities. PCC means a shift away from the traditional focus on the patient as a passive recipient of medical treatment to a more collaborative view of health care where patients (often with relatives) are engaged as active partners in the care planning and decision-making process. Establishing a collaborative and transparent partnership is crucial based on ethical principles and mutual respect [52, 53].

#### Study strengths and limitations

This study’s main strength is its exploration of an understudied area: experiences of support in people with stress-related exhaustion in the early stages of sick leave. We used the phenomenological hermeneutic approach in order to interpret complex experiences through a comprehensive analysis. The study included 12 participants’ narratives which may seem to be small but in qualitative interviews the credibility lies in each interview’s quality rather than the number of samples. The interview data in this study were rich and provided more profound insight into the meaning of support for people living with stress-related exhaustion. Moreover, we have reached variation by interviewing women and men of different ages, and with various professions and socioeconomic backgrounds. We used follow-up questions tailored specifically to each narrative to clarify the findings. Regarding dependability, the participants were diagnosed with the same condition by primary care physicians, and the same person conducted the interviews, and used the same interview guide. Moreover, the interpretation of the texts was undertaken by several researchers to ensure the trustworthiness of the findings, and which are presented with representative quotations [20, 54]. However, phenomenological hermeneutics does not seek one universal truth as there is always more than one way to analyse and interpret the data; the result of this study represents one of several possible interpretations [39]. Not all possible interpretations are equally probable, and to reach a trustworthy interpretation, all authors contributed to a systematic reflective discussion during each step of the analysis until consensus was reached [40].

### Conclusion and clinical implications

The findings from this study indicate that people affected by stress-related exhaustion need allies from their surrounding networks in their struggle to maintain their dignity. Commitment, mutual respect, and shared understanding were described by participants as essential components of such support. Our findings highlight that, when being exhausted, the affected person needs to be able to take the back seat without feelings of shame and guilt. Possible implications for clinical practice, deriving from the findings, suggest that when encountering patients with stress-related exhaustion, professionals firstly need to meet patients as persons beyond their diagnosis and taking their experiences and current situation into account. Moreover, professionals need to acknowledge the severity and the complexity of the condition and the importance of professionals’ personal and emotional engagement in the recovery process. The findings also point to the importance of recognizing patients as persons and establishing a partnership between health care professionals and patients (often with close ones). This approach is emphasized in person-centred care, where appropriate support is based on patient’s needs, resources, and situation. Such a partnership may avoid violating the dignity of people with stress-related exhaustion and instead improve their health and optimize their recovery.

## Supporting information

S1 File (DOCX)
