# Supplementary material for: Having allies—Experiences of support in people with stress-related exhaustion: A qualitative study
Source: PLoS One. 2022 Nov 9;17(11):e0277264. doi: 10.1371/journal.pone.0277264 (PMC9645633; doi:10.1371/journal.pone.0277264)
Supplement: S1 File — (DOCX) [file pone.0277264.s001.docx]

**Interview guide**

Experiences of support in patients with stress-related exhaustion

**Overall question:**

-How do you experience support in everyday life (when being affected by stress-related exhaustion)?

**Probing questions supporting narration**

What do you mean by that?

How did that make you feel?

What do you think about that?

How did that affect you?

**Additional questions**

- What does support mean to you in your situation?

-Is there anything you would like to add?
